# Impact of bacterial aerosol, particulate matter, and microclimatic parameters on animal welfare in Chorzów (Poland) zoological garden

**DOI:** 10.1007/s11356-020-10680-9

**Published:** 2020-09-11

**Authors:** Jacek Grzyb, Krzysztof Pawlak

**Affiliations:** 1 Department of Microbiology and Biomonitoring, University of Agriculture in Kraków, Mickiewicza Ave 24/28, 30-059 Kraków, Poland; 2 Department of Zoology and Animal Welfare, University of Agriculture in Kraków, Mickiewicza Ave 24/28, 30-059 Kraków, Poland

**Keywords:** Bioaerosol exposure, Occupational exposure, Bacterial aerosol, Health risk, Particulate matter

## Abstract

Zoos are very popular facilities visited by entire families with children, who come there to watch live animals. Zoos also provide workplaces for a large number of people directly looking after the animals. For places designed to house animals, regardless of whether they are farm animals, pets, or zoo animals, a higher concentration of both dust and potentially harmful bioaerosols can be expected. Unfortunately, there are almost no studies concerning the concentration of bacterial bioaerosols and particulate matter in animal shelters that would answer the question whether the level of these pollutants is constant or variable and dependent on a particular zoo, group of animals, their number in enclosures, or season. This study aimed to assess the levels of bacterial aerosol in rooms intended for animals (giraffes, camels, elephants, kangaroos, and colobinae) in the Silesian Zoological Garden in Chorzów (Poland). The bioaerosol samples were collected using a six-stage Andersen cascade impactor to assess the concentrations and size distribution of airborne bacteria. Particulate matter (PM10) was assessed using an electronic dust meter. Measurements of microclimate parameters were carried out using the Airflow™ Instruments Velocity Meter TA440, while gas concentrations were determined applying GFG Microtector II G450. The results showed that the concentration of airborne bacteria varied significantly between facilities for the analyzed animal groups. The lowest concentration of the total bacterial aerosol was observed in enclosures for colobinae (approx. 850 CFU/m^3^), while the highest—in rooms for elephants (approx. 105,600 CFU/m^3^). The average share of respirable fraction of bacteria was quite high, with values ranging from 62.9 (colobinae) to 86.9% (elephants), indicating potential harmfulness to the health of exposed people. PM10 concentrations were relatively low (10–86 μg/m^3^) and did not exceed the limit values for occupational exposure. Moreover, the levels of bacterial bioaerosol in almost all cases did not exceed the limit values. As the animals constitute a significant source of bioaerosol, attention should be paid to thorough cleaning of animals and their shelters, as well as maintaining appropriate levels of microclimate parameters in the facilities.

## Introduction

Zoological gardens create opportunity for saving animal species threatened with extinction, what helps to preserve biodiversity and protect genetic resources. The history of zoos is very long and it dates back to ancient times. However, it was only during the last 100 years that the conditions in zoos changed as a result of the development of veterinary medicine, as well as providing animals with better living conditions and adequate food allowing for a significant extension of animal life (Tombarkiewicz et al. 2008). However, the presence of microorganisms in animal rooms remains unchanged.

Ensuring animal welfare requires providing animals with housing that is necessary either only during cold seasons or throughout the entire year, depending on animal species. Securing animal well-being involves maintaining appropriate room parameters: adequate temperature and humidity and proper lighting, as well as sufficient surface and cubature (Pawlak et al. 2008; Kruszewicz 2011). The EU Council Directive 1999/22/EC requires zoos to guarantee animal welfare, what is a fundamental prerequisite for their operation. It involves providing the so-called Five Freedoms, i.e., animal freedom from pain, injuries and illness, hunger and thirst, fear and stress, and discomfort and unnatural behavior caused by lack of space (Webster 1994). Ensuring proper conditions in zoological gardens is also required by legal regulations included in the Regulation of the Minister of Environment of 2004 (Journal of Laws 2004).

Creating proper hygienic conditions is associated with meeting animal welfare assumptions. The animals themselves constitute a basic source of microorganisms in the rooms where they stay. Other sources include bedding, food residues, feces, and urine (for part of the day). The microclimatic conditions in the rooms convenient for animals are also optimal for microbes; thus, they can multiply freely. The activity of animals creates favorable conditions for generating organic dust particles. Microbial cells are usually associated with dust particles, what facilitates penetration of microorganisms with the air (bacteria—in the form of cells and spores, molds as spores). Previous research has shown that microbial aerosol concentrations were relatively high in the animal facilities (Sowiak et al. 2012; Grzyb and Lenart-Boroń 2019). The concentrations of airborne bacteria in rooms intended to house animals are usually much higher than in human dwellings.

High concentrations of bioaerosol in animal rooms are accompanied with dust as well odorous volatile compounds. These parameters are usually correlated with the size of the animals and the stocking (Millner 2009).

There are studies demonstrating the relationship between internal environment pollution and the occurrence of acute and/or chronic adverse health effects and diseases (Kaliste et al. 2002; Bouillard et al. 2005; Samadi et al. 2013). Respiratory system problems (e.g., asthma rhinitis, sinusitis, bronchitis) occur most often. Other problems include allergic mechanisms, fatigue, headache, and gastrointestinal distress (Douglas et al. 2018). These problems can affect both animals and zookeepers working with them, who feed animals and perform daily care activities (Lorenz 2004).

In addition to animals and zookeepers, visitors, especially small children, are exposed to the impact of bioaerosols within zoo premises. Higher exposure of children results from a different structure of their respiratory tract than in adults, their greater mobility, and inhalation of more air in relation to their mass and not fully mature immune system (Yoon et al. 2011).

The main aim of the research was to assess the airborne bacteria and dust pollution of air in combination with zoohygienic parameters in the selected animal enclosures in the Silesian Zoological Garden in Chorzów. The specific aims covered the determination and analyses of the following:

- whether the total (TC) and respirable fractions (RF) of bacterial bioaerosol vary depending on the animals tested;
- the size distribution of bacterial bioaerosol and dust particles in rooms intended for five various animal species;
- which factors most affect the concentration of bacterial bioaerosol;
- whether the observed bioaerosol concentrations may pose health risks to the zoo workers and visitors;
- whether microclimatic factors, tested gasses, and PM10 affect bacterial aerosol concentration.

The obtained results will allow to determine the air quality in zoological gardens and the potential health risks associated with the contact with bioaerosol and dust particles present in zoos. These studies are a continuation of research performed previously in the Zoological Garden in Kraków.

## Materials and methods

The study was conducted in the Silesian Zoological Garden in Chorzów (Poland) established in 1958. It is a medium-sized zoo, with an area of 47.62 ha. The facility is located on a flat area, 272 m above sea level.

The measurements were carried out inside five facilities where the following animals are kept: giraffes (*Giraffa camelopardalis reticulata*), elephants (*Elephas maximus*), camels (*Camelus bactrianus*), colobinae (*Colobus guereza*), and kangaroos (*Macropus rufus*).

The selection of animal species for the study was based on the size of animals—large (giraffes, elephants, camels) versus small (colobinae and kangaroos).

The age of animal houses constituted another selection criterion—giraffes and colobinae were kept in new enclosures, while elephants, camels, and kangaroos in older ones.

One site located outdoors, on the parking at a distance of approx. 5 m from the front of the office building, served as a control.

**Fig. 1:**
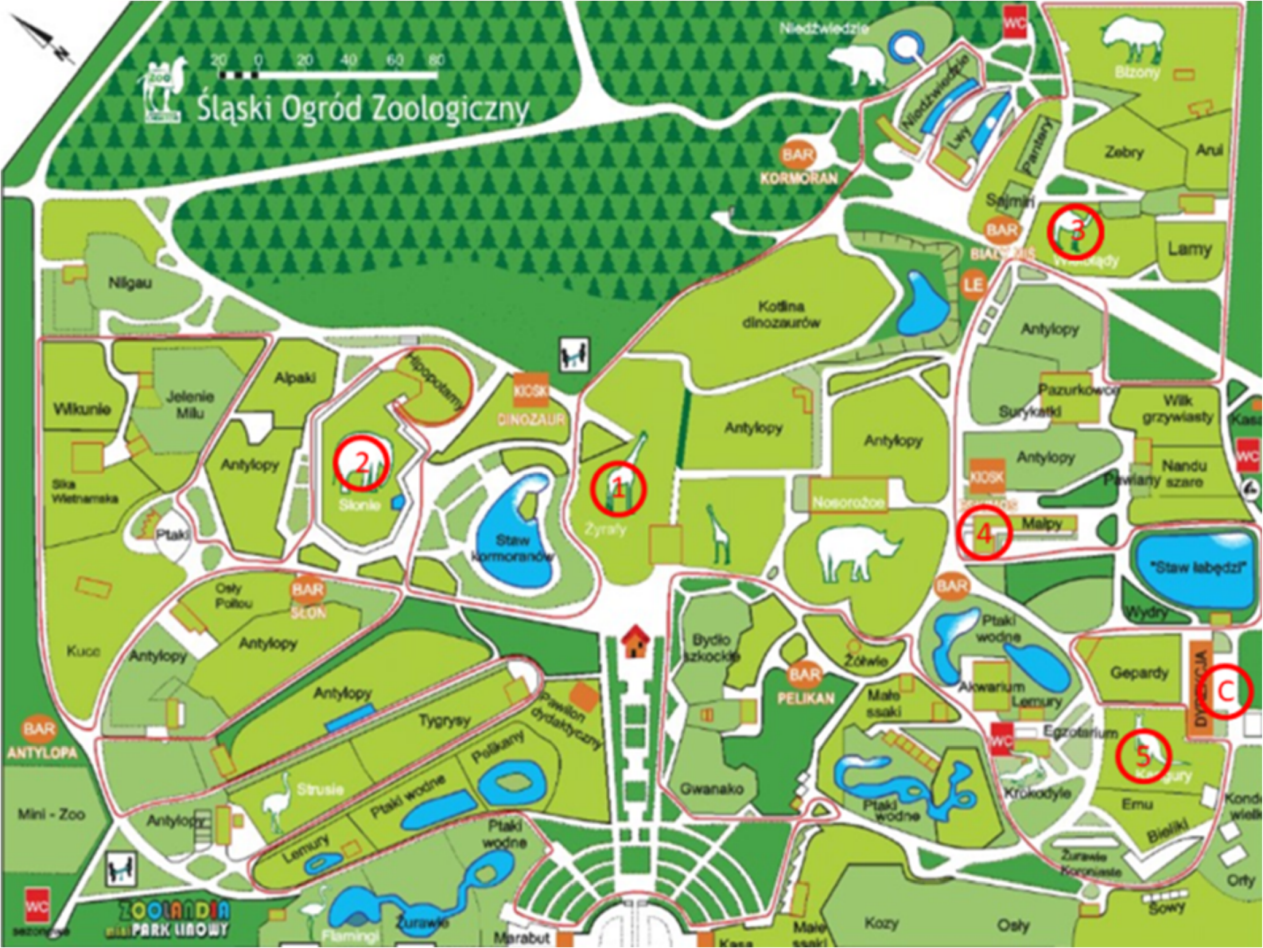
Location of the sampling sites

The location of the sampling sites is shown in Fig. 1 and their characteristics in Table 1.

**Table 1:** Characteristics of the studied shelters

Parameter	Group of animals				
	Elephants (*Elephas maximus*)	Camels (*Camelus bactrianus*)	Colobinae (*Colobus guereza*)	Giraffes (*Giraffa camelopardalis reticulata*)	Kangaroos (*Macropus rufus*)
Total area (m^2^)	200	50	38	343	25
Year of construction	1960s	1960s	2010	2013	1960s
Number of animals	2	2	10	5	5
Area per 1 animal (m^2^)	100	25	3.8	68.6	5
Type of ventilation	Lack	Lack	Mechanical	Mechanical	Lack
Mean animal weight (kg)	3500	480	14	1500	47
Ratio—kg of animal weight per 1 m^2^ of area	35	19.2	3.7	21.9	9.4
Type of litter	Lack	Sawdust beech	Lack	Sawdust beech	Sawdust beech

The air samples were collected in the period of 1 year using a six-stage Andersen-Graseby model WES-710 cascade impactor (Westech Instrument, UK). The use of a cascade impactor allows the separation of bioaerosol into fractions based on the aerodynamic diameter of its particles: above 7 μm (stage one), 4.7–7 μm (stage two), 3.3–4.7 μm (stage three), 2.1–3.3 μm (stage four), 1.1–2.1 μm (stage five), and 0.65–1.1 μm (stage six). Aggregates containing microorganisms with aerodynamic diameters below 4.7 μm are treated as the respirable fraction of bioaerosol.

Samples were collected between 10.00 AM and 1.00 PM. Six Petri dishes were used to collect the sample—one for each impactor stage. Between each sampling, swabs soaked in 70% isopropanol were used to disinfect the impactor. For sampling time from 20 to 180 s and the air flow through the impactor amounting to 28.3 l/min, the volume of aspirated air reached the value between 9.4 and 84.9 l. Sampling time depended on the predicted bacterial concentration at the locations. Air samples were taken at a height of 1.5 m above ground level, which corresponds to the location of the human breathing zone. Microbial medium trypticase soya agar (TSA – Biomaxima Poland) for the total number of bacteria was used to collect bioaerosol samples. The plates were incubated at 37 °C for 1 day, then at 22 °C for the next 3 days and at 4 °C for another 3 days in aerobic conditions. After incubation time, the colonies of bacteria were counted and the results were expressed using commonly applied method for estimating the concentration of living microorganisms, i.e., the number of colony-forming units per m^3^ of air (CFU/m^3^). This is due to problems with direct measurement of concentrations of viable airborne bacteria. The results from three replicates were used to calculate mean values, which are shown as final results in figures and tables.

At the same time, microclimatic measurements (air temperature, relative humidity, and air movement) were carried out using the Airflow™ Instruments (USA) Velocity Meter TA440. PM10 dust concentrations were determined using university measuring stations (UMS, serial no. U32 and U33, Poland), developed within the framework of the Storm&DustNet project implemented at the Jagiellonian University in Krakow, while gas concentrations (carbon dioxide, ammonia, and hydrogen sulfide) using GFG Microtector II G450 (Germany).

The measurement devices (the Andersen impactor, Airflow™ Instruments Velocity Meter TA440, GFG Microtector II G450, electronic dust meter) and all laboratory equipment used in our study hold valid certificates and are regularly checked. Tests were carried out based on Polish standard PN-EN 12322 (2005) which meant applying quality control procedures, such as an adequate number of culture media, plates, and replicates for each series of measurements and standard ISO 11133:2014-7.

There are no relevant standards for bioaerosol in animal housing; therefore, the bioaerosol concentrations obtained during the study were referred to the proposals of the Team of Experts in Biological Factors (Polish: ZECB) (Augustyńska and Pośniak 2016), treating animal rooms as working premises contaminated with organic dust (Table 2).

**Table 2:** Proposals for acceptable concentrations of airborne microorganisms in the working environment according to the Team of Experts in Biological Factors (ZECB)—the values applicable in Poland

Microbiological agent	Acceptable concentration (CFU/m^3^)
Mesophilic bacteria (total count)	100,000
Mesophilic bacteria (respirable fraction—RF)	50,000

Statistical analysis was performed using the Statistica 13 software (StatSoft, USA). The observed values of bacterial aerosol are presented as average values with standard deviation and ranges. The normality of data distribution was tested using the Shapiro-Wilk test. The distribution of total (TC) and respirable (RF) fraction of bioaerosol values was close to normal and other data were not normally distributed; therefore, both parametric (a one-way ANOVA, followed by the post-hoc Tukey’s test) and non-parametric (the Kruskal-Wallis test) tests were applied to assess the significance of differences between the concentrations of bioaerosols in rooms for different animals. To assess whether there are statistically significant relationships between the concentrations of bioaerosol components and other analyzed parameters in the tested site, the Pearson’s correlation coefficients were applied.

## Results

Table 3 presents data on the total concentration of bacteria and respirable fraction expressed as means along with standard deviations and concentration ranges. The lowest average concentrations and standard deviations for both total count (TC) and respirable fraction (RF) were found in rooms for colobinae (2178 ± 2015 CFU/m^3^ and 1371 ± 1363 CFU/m^3^, respectively).

**Table 3:** Average, standard deviation, and range of bacterial bioaerosol in animal premises in Chorzów zoological garden

Group of animals	Fractions of bioaerosol (CFU/m^3^)	
	Total count of bacteria (TC) average ± st. dev. (range)	Respirable fraction (RF) average ± st. dev. (range)
Giraffes	17,727 ± 17,435 (1978–41,288)	12,725 ± 12,847 (1837–30,825)
Elephants	31,617 ± 49,427 (3533–105,576)	27,476 ± 43,529 (3344–92,644)
Camels	11,173 ± 7498 (1554–17,675)	9511 ± 6981 (1272–16,048)
Colobinae	2178 ± 2015 (847–5182)	1371 ± 1363 (471–3391)
Kangaroos	14,137 ± 8281 (2403–20,149)	9985 ± 6917 (989–17,604)
Control	416 ± 255 (223–791)	276 ± 236 (118–622)

Approximately 5 times lower concentrations were found in the control area (TC: 416 ± 255 CFU/m^3^; RF: 276 ± 236 CFU/m^3^). The highest bacterial aerosol concentrations were found in the enclosures for elephants (TC: 31,617 ± 49,427 CFU/m^3^, RF: 27,476 ± 43,529 CFU/m^3^). They were 15 times higher for TC and 20 times higher for RF compared with premises for colobinae. As compared with the control, these concentrations were 76 times higher for TC and 100 times higher for RF. Very similar concentrations of bioaerosol were recorded for camels and kangaroos.

**Fig. 2:**
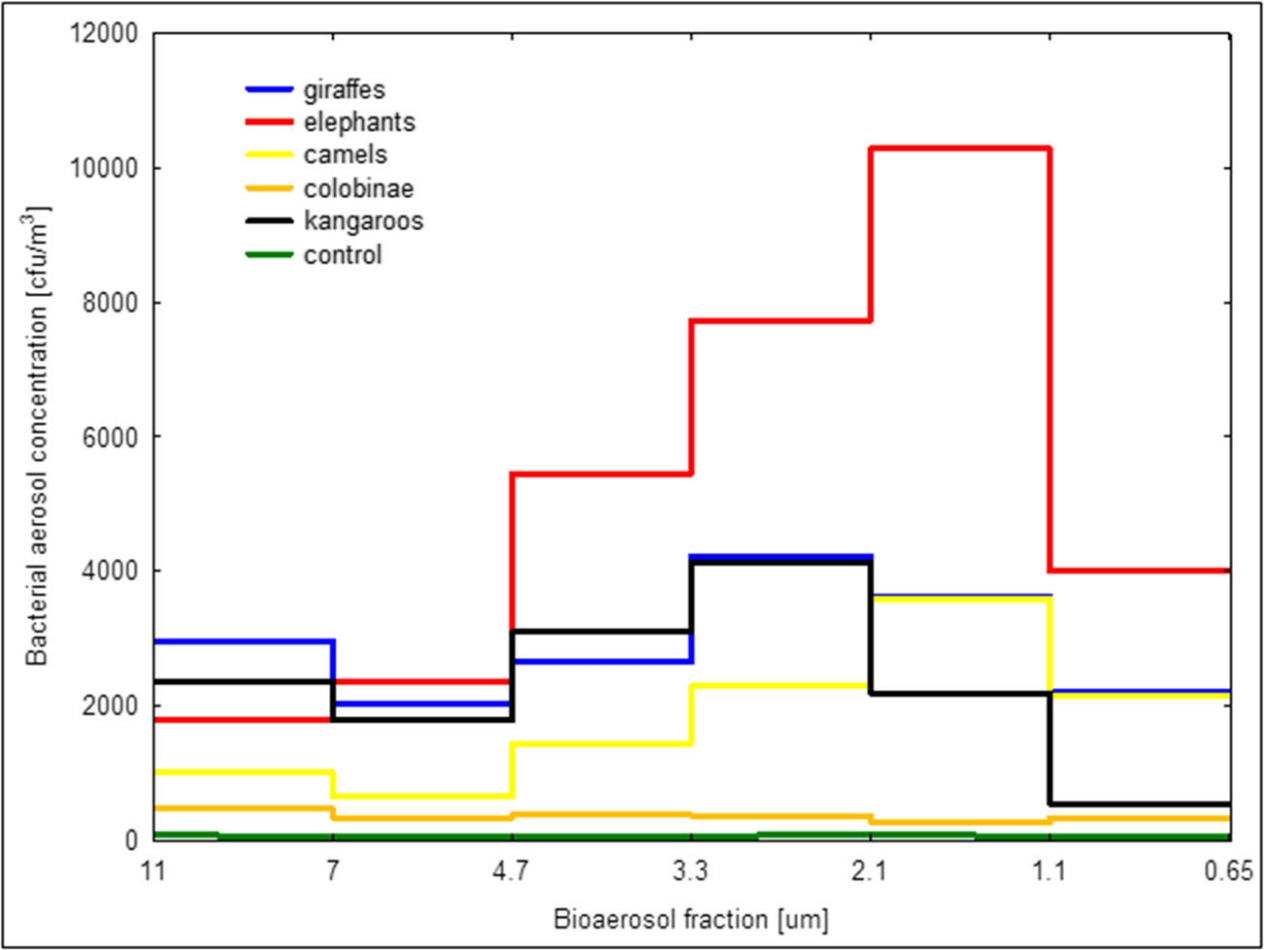
Average concentrations of bacterial bioaerosol fractions

**Fig. 3:**
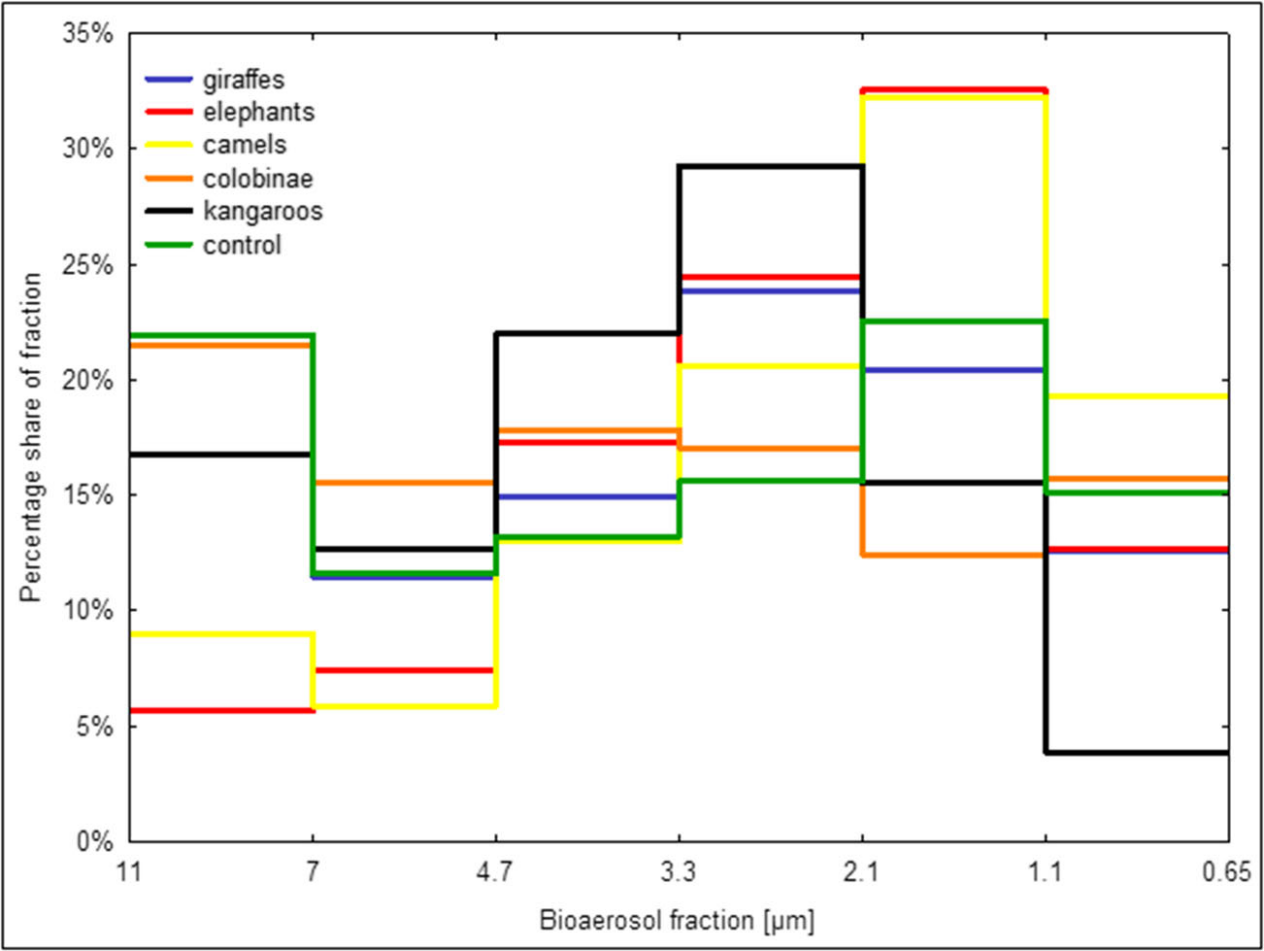
The share of bacterial bioaerosol fractions for tested animals (%)

The use of a cascade impactor for this research allowed an estimation of the concentration of a particular bioaerosol fraction (Fig. 2) and the share of bioaerosol fractions for different animals (Fig. 3). The results obtained in the facilities for elephants differ strongly (Fig. 2)—all concentrations from the respirable fraction were significantly higher than in the case of other animals and were in the range from 3997 to 10,287 CFU/m^3^. The lowest concentrations of bioaerosol were observed in colobinae shelters, for the respirable fraction (< 4.7 μm) concentrations ranged between 271 and 389 CFU/m^3^. In comparison with the control, bioaerosol concentrations in animal houses were on average 5 (colobinae) to 76 times higher (elephants).

**Fig. 4:**
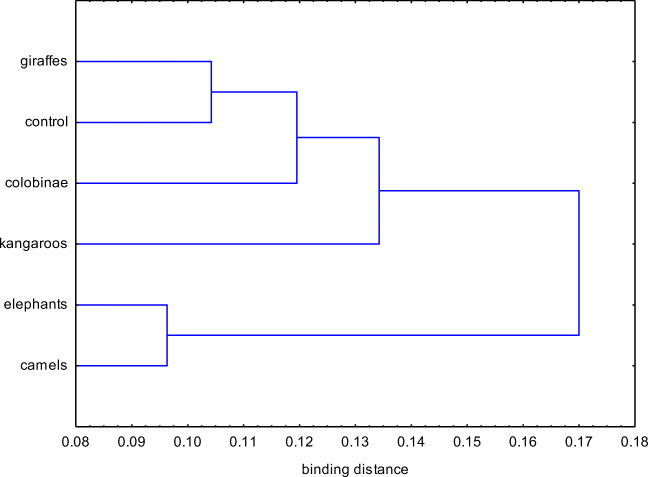
Cluster analysis for shares of fractions of bacterial bioaerosol in animal shelters in the zoo in Chorzów

Respirable fraction (RF) in the range of 2.1–1.1 μm had the largest share in the total concentration of bacterial bioaerosol (Fig. 3), what is especially visible in the case of the shelters for elephants and camels (32.5% and 32.2% respectively). Bioaerosol particles from this fraction end up in the alveoli. The fraction 3.3–2.1 μm was not much smaller, especially in the kangaroo room (29.3%). The bioaerosol from this fraction reaches secondary bronchi. Taking into consideration all examined sites, the share of 7–4.7 μm fraction was the smallest, while in the case of shelters for a specific group of animals, the smallest share was found for the 1.1–0.65 μm fraction (3.9%) in the kangaroo enclosures. After cluster analysis (Fig. 4), it can be estimated that shelters for elephants, camels, giraffes, and a control site have the closest percentage shares in bacterial bioaerosol fractions. The average share of the respirable fraction of bioaerosol (RF) in total concentration (TC)—in annual terms—ranged from 62.9% in the case of facilities for colobinae to 86.9% in the case of rooms for elephants (Table 4). However, taking into consideration the share of respirable fraction depending on the season—the spread of RF share in TC is significant and in the case of shelters for kangaroos, it amounted to 112%—between the winter (87.4%) and the spring (41.2%), while for shelters for elephants, it was only 22% (summer—77.5%; winter—94.7%).

**Table 4:** Percentage share of RF in the season (%)

Season	Giraffes	Elephants	Camels	Colobinae	Kangaroos	Control
Spring	62.3%	87.8%	82.0%	55.6%	41.2%	38.5%
Summer	92.9%	77.5%	81.8%	47.4%	47.1%	57.9%
Autumn	74.7%	81.5%	69.8%	73.7%	84.6%	69.2%
Winter	75.7%	94.7%	97.0%	65.5%	87.4%	78.6%
Average	71.80%	86.90%	85.10%	62.90%	70.70%	66.50%

**Fig. 5:**
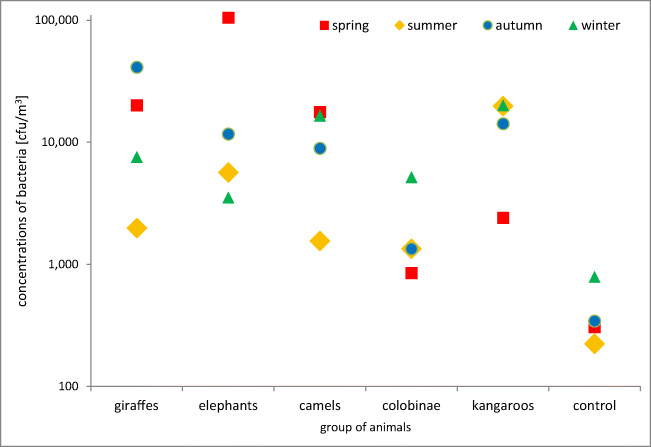
Total bioaerosol concentration in animal shelters depending on the season

Conducting research throughout the entire year enabled the comparison of the bioaerosol concentrations in different seasons (Fig. 5). For 3 groups of tested animals (giraffes, elephants, and camels), the highest bacterial aerosol concentrations were found during the spring. For the comparison in the case of colobinae, kangaroos, and control, the highest concentrations were recorded during winter. Taking into account all measurements taken, the permissible concentration of bioaerosol (assumed based on *Proposals for acceptable concentrations of airborne microorganisms in the working environment according to the Team of Experts in Biological Factors*)—both in terms of the total concentration of bacterial aerosol and the concentration of respirable fraction—was exceeded only once during the spring in enclosures for elephants (Table 2).

Table 5 shows the dependence of the I/O ratio (that is the relationship of indoor bioaerosol concentration in shelters for animals to outdoor concentration called control) on the group of animals tested and the season for TC and RF. The lowest I/O ratio was obtained in shelters for colobinae, and the highest in enclosures for giraffes and elephants.

**Table 5:** Dependence of ratio I/O on the group of animals/season for total concentration (TC) and respirable fraction (RF) of bacterial bioaerosol

Group of animals/season	I/O for TC	Group of animals/season	I/O for RF
Colobinae/spring	3	Colobinae/spring	4
Colobinae/autumn	4	Colobinae/autumn	4
Elephants/winter	4	Colobinae/summer	5
Colobinae/summer	6	Elephants/winter	5
Colobinae/winter	7	Colobinae/winter	5
Camels/summer	7	Kangaroos/spring	8
Kangaroos/spring	8	Giraffes/winter	9
Giraffes/summer	9	Camels/summer	10
Giraffes/winter	10	Giraffes/summer	14
Camels/winter	21	Camels/winter	26
Elephants/summer	25	Camels/autumn	26
Kangaroos/winter	25	Kangaroos/winter	28
Camels/autumn	26	Elephants/summer	34
Elephants/autumn	34	Elephants/autumn	40
Kangaroos/autumn	41	Kangaroos/autumn	51
Camels/spring	58	Kangaroos/summer	72
Giraffes/spring	65	Giraffes/spring	106
Kangaroos/summer	88	Camels/spring	123
Giraffes/autumn	120	Giraffes/autumn	130
Elephants/spring	344	Elephants/spring	785

The measured microclimatic parameters and PM10 concentration (average ± st. dev and range) are presented in Table 6. The lowest average temperatures were recorded in rooms for camels (15.8 °C, range from 7 to 22 °C). These temperatures were probably affected by the largest air movement (0.61 ± 0.38 m/s) causing cooling of both facilities and bodies of animals staying inside. This is due to the fact that the room door was often open. The highest temperatures with the smallest range were recorded in shelters for giraffes (21.4 °C, range: 19.8–22 °C). The average temperatures in animal rooms were 2.4 to 3.3 times higher than the average for the control. The average relative humidity of the air in animal housing rooms ranged between 54.5 and 78.7%, with the range 35–87%. In the control, the humidity range was smaller, from 49 to 73%.

**Table 6:** Microclimatic parameters and dustness (PM10) in the animal shelters at the zoo in Chorzów

Animals	Temperature (°C)	RH (%)	Air flow (m/s)	PM10 (μg/m^3^)
	Average ± st. dev. (range)			
Giraffes	21.4 ± 1.07 (19.8–22)	54.5 ± 17.25 (35–70)	0.25 ± 0.13 (0.08–0.41)	42 ± 29.58 (22–86)
Elephants	18.8 ± 3.08 (16–23.2)	71 ± 6.48 (64–79)	0.13 ± 0.05 (0.08–0.2)	36.2 ± 27.80 (10–75)
Camels	15.8 ± 6.41 (7–22.2)	66.7 ± 14.66 (55–87)	0.61 ± 0.38 (0.38–1.19)	32.2 ± 25.26 (12–69)
Colobinae	19.3 ± 2.52 (16–22)	78.7 ± 6.65 (69–84)	0.30 ± 0.32 (0.07–0.77)	36.7 ± 30.71 (14–82)
Kangaroos	19.7 ± 2.15 (16.8–22)	65 ± 3.82 (60–68)	0.15 ± 0.11 (0.02–0.26)	30.7 ± 24.51 (10–66)
Control	6.55 ± 5.89 (0.2–14.2)	59.2 ± 10.21 (49–73)	1.15 ± 0.74 (0.14–1.81)	54.2 ± 40.5 (18–112)

The average level of PM10 concentration (Table 6) in all animal rooms was lower than in the control (30.7–42 μg/m^3^ versus 54.2 μg/m^3^). The range in animal facilities amounted to 10–86 μg/m^3^, and in the control to 18–112 μg/m^3^. The lowest average PM10 concentration was found in shelters for kangaroos and the highest in enclosures for giraffes.

Gas concentrations (CO_2_, NH_3_, and H_2_S) measured in the tested buildings are presented in Table 7. The average CO_2_ concentration in animal shelters was 2 to 5 times higher compared with the control. No hydrogen sulfide was reported, while low ammonia was found only in shelters for giraffes.

**Table 7:** Gas concentrations in animal shelters at the zoo in Chorzów

Animals	Conc. CO_2_ (ppm)	Conc. NH_3_ (ppm)	Conc. H_2_S (ppm)
	Average ± st. dev. (range)		
Giraffes	2375 ± 394.75 (1800–2700)	1.75 ± 3.5 (0–7)	0 ± 0 (0–0)
Elephants	1475 ± 457.34 (1000–2000)	0 ± 0 (0–0)	0 ± 0 (0–0)
Camels	850 ± 288.67 (500–1200)	0 ± 0 (0–0)	0 ± 0 (0–0)
Colobinae	1750 ± 858.29 (1000–2800)	0 ± 0 (0–0)	0 ± 0 (0–0)
Kangaroos	747.5 ± 212.19 (490–1000)	0 ± 0 (0–0)	0 ± 0 (0–0)
Control	437.5 ± 50.57 (380–500)	0 ± 0 (0–0)	0 ± 0 (0–0)

The Pearson’s correlation analysis was performed for all parameters tested (Table 8). This analysis shows that statistically significant positive relationships occur between all bioaerosol fractions including TC and RF. Negative relationships occur between the temperature and the concentration of PM10, i.e., an increase in temperature causes a decrease in the concentration of PM10. On the other hand, strong air movement reduces the concentration of the “thickest” bioaerosol fractions (11–7 μm and 7–4.7 μm) and leads to a decrease in the temperature in animal facilities.

**Table 8:** correlation between the tested parameters

	F1	F2	F3	F4	F5	F6	TC	RF	PM10	T	RH	Air flow	CO_2_	NH_3_	H_2_S
F1															
F2	0.88														
F3	0.69	0.89													
F4	0.70	0.89	0.94												
F5	0.59	0.78	0.85	0.92											
F6	0.67	0.78	0.72	0.77	0.80										
TC	0.77	0.92	0.94	0.96	0.90	0.85									
RF	0.68	0.87	0.92	0.96	0.95	0.88	0.98								
PM10															
T									− 0.47						
RH															
Air flow	− 0.54	− 0.44								-0.47					
CO_2_															
NH_3_															
H_2_S															

F1–F6—fractions of bioaerosol: F1 = 11–7 μm, F2 = 7–4.7 μm, F3 = 4.7–3.3 μm, F4 = 3.3–2.1 μm, F5 = 2.1–1.1 μm, F6 = 1.1–0.65 μm

## Discussion

Zoological gardens represent one of the most interesting objects that are utilized as tourist and educational facilities both for children and adults. The number of people visiting zoo premises reaches dozens or even hundred thousands annually—per one zoo. If we take into account employees directly involved in looking after the animals, this number would be even higher. The last group is not so large but has contact with animals for few hours daily throughout the entire year. Both tourists visiting zoos and animal keepers are exposed to aerosols produced by animals—including bioaerosols and dust aerosols. However, the number of studies dealing with this issue is very scarce.

The threshold values concerning aerosol harmfulness were determined by the Team of Experts in Biological Factors (Polish: ZECB). Concentration levels for harmful biotic agents that are usually present in various types of compartments were established based on the large amount of results. A potential threat posed by a given agent was also taken into consideration. These data were used to determine concentrations typical for a given type of room. When the critical threshold is exceeded, it means that some unusual, potentially harmful source of pollution has appeared. Due to considerable methodological difficulties, these recommendations apply solely to two types of compartments—residential buildings and public utility facilities (with lower acceptable concentrations of bioaerosols) and work premises polluted with organic dust (Górny et al. 2011; Górny 2019).

The threshold values for bacterial bioaerosol concentrations recommended by ZECB, amounting to 1 × 10^5^ CFU/m^3^ and 5 × 10^4^ CFU/m^3^ of air for TC and FR, respectively, in most cases were not exceeded in analyzed animal enclosures. Acceptable concentration was exceeded only once in rooms housing elephants (for TC and RF).

The comparison of the research results obtained in the course of the examinations conducted in the zoo in Kraków (Grzyb and Lenart-Boroń 2019) with those delivered as a result of measurements taken in Chorzów revealed that concentrations recorded in the shelters for giraffes in Kraków zoo were 12 times lower, while in rooms for monkeys about 3 times higher. Due to the fact that the facilities for giraffes in both zoos are of the same age, the difference in bioaerosol concentration may be the result of different area per one animal (Kraków: 244 m^2^; Chorzów: 66.8 m^2^). On the other hand, in the case of monkeys, the difference in bioaerosol concentration may be the effect of the type of litter used—sawdust beech in the zoo in Kraków, no litter in Chorzów. As revealed by Samadi et al. (2012), the type of litter used in facilities intended for animals represents a critical factor that determines not only the level of bacterial and fungal bioaerosols but also the concentration of dust and endotoxins. The lowest dust concentrations were recorded in animal rooms with no litter, followed by those with sawdust bedding.

The differences in the concentration of bacterial bioaerosol in the shelters designed to house animals were demonstrated earlier by many authors (Matković et al. 2006; Millner 2009; Samadi et al. 2012; Zhao et al. 2014; Grzyb and Lenart-Boroń 2019; Islam et al. 2019, 2020). These differences depend on many factors, which are either directly connected with animals (species, size, age, and physical activity) or broadly defined animal environment that includes the size and volume of the facilities, type of ventilation, and animal density. Microclimatic conditions also provide contribution. The concentrations of bacterial bioaerosol recorded in the zoos are significantly lower in comparison with those detected on large-scale farms. The research carried out by Popescu et al. (2008) in cowsheds in Romania showed variations in bacterial concentration ranging from 7.9 × 10^4^ to 3.5 × 10^5^ CFU/m^3^. On the other hand, Masclaux et al. (2013) reported that in the pig houses in Switzerland, bacterial concentration was in the range from 1.6 × 10^5^ to as much as 6.1 × 10^10^ CFU/m^3^. However, the concentrations of bacterial bioaerosols recorded in animal enclosures in the zoo in Chorzów are higher than those determined for indoor environments. Pastuszka et al. (2000) estimated that in residential buildings, it amounts to 1 × 10^3^ CFU/m^3^ on average, while in the offices, it was even lower and reached the value of approximately 3 × 10^2^ CFU/m^3^. Similar concentrations in housing premises in South Korea were reported by Moon et al. (2014) and, depending on the season, ranged from 3 × 10^2^ CFU/m^3^ (winter) to 7 × 10^2^ CFU/m^3^ (autumn).

Islam et al. (2019) emphasize the heterogeneity of aerosol generated in animal houses that is mostly of organic origin (approximately 90%) and includes particles of different shape, size, and density.

The application of a six-stage Andersen impactor enabled analysis of the size distribution of bioaerosol particles at the studied locations expressed as its aerodynamic diameter. It allows to establish the potential depth of bioaerosol penetration into the human respiratory tract for particular bioaerosol fractions (Górny et al. 2016). Another important feature of this method is connected with the possibility to determine the share of respirable fraction (RF) in the total concentration (TC) of bacterial bioaerosol. The respirable fraction of bioaerosol includes particles smaller than 4.7 μm, but the particles below 2.5 μm pose the most considerable threat to the health of the people exposed. They may penetrate into the lower respiratory tract (bronchioles and alveoli), where they produce allergic and toxic effects, causing multiple pulmonary and cardiovascular disorders as well as cancer, low birth mass, and premature death (Owen et al. 1992; Pastuszka et al. 2000; Samadi et al. 2013; Morakinyo et al. 2016).

In the present study, taking into account the size distribution of bioaerosol fractions, significant differences between rooms for various animals were found (Fig. 2, Table 4). The share of RF in the total concentration of bacterial bioaerosol was in the range between 41.2 and 97%, with the mean value of 74%. The share of RF in our research is high. It is extremely important to remember that the particle size of bioaerosols is strictly associated with its penetrability into the human respiratory system. The more small particles are present in the air, the greater the share of respirable fraction in the total concentration of bioaerosol. It regards animals, employees, and zoo visitors to the same extent. Brągoszewska et al. (2018) suggested that RF exceeding 80% should be treated as potentially harmful to the people exposed. However, there are studies demonstrating that people growing up on farms among animals in the environment with high dust and bioaerosol concentrations are less prone to develop allergy, atopic dermatitis, and atopic asthma (Kullman et al. 1998).

In the present study, the fraction with particle size 2.1–1.1 μm had the largest share (12.4–32.5%), depending on the animal group, while the lowest share was reported for fraction 7–4.7 μm. In the studies performed at the zoo in Kraków (Grzyb and Lenart-Boroń 2019), the fraction with a larger aerodynamic diameter had the largest share (3.3–2.1 μm versus 2.1–1.1 μm), but the participation of this fraction in TC was very similar. The fraction 7–4.7 μm had the smallest share, similarly to the current study. Moreover, during studies in both zoos, it was found that the respirable fraction constituted 60.1–90.2%. The research undertaken by Brągoszewska and Pastuszka (2018) concerning atmospheric air in the city center revealed that particles with even greater aerodynamic diameter have the largest share (4.7–3.3 μm). During the spring and summer, this share was the highest and exceeded 35%. Lis et al. (2008) observed that the share of RF of bacteria for farmhouses ranged from 38 to 80%, with the mean value amounting to 55%. As compared with this study, the share for RF had narrower range, while the average RF value was higher by more than 20%. A very high proportion of RF in bacterial bioaerosol ranging from 83.5 to 88.0% was observed by Chien et al. (2011), who carried out bioaerosol measurements in chicken and swine houses. Moreover, RF in these studies was characterized by very low variability.

Our observations indicate that the share of respirable fraction is affected by the size of animals or their species. Larger animals and those standing on the litter (giraffes, elephants, and camels) have a higher proportion of RF in the TC of bacterial bioaerosol. This may be due to the fact that as they move, they build up the dust and their weight breaks down the aggregates into smaller fragments.

Another useful indicator that can be applied in environmental studies is represented by the ratio of indoor microbiological air pollution to outdoor air pollution (I/O ratio). It is an extremely valuable tool for verifying the presence of an internal source of microorganism emission (American Industrial Hygiene Association 1989). When the I/O is higher than 1, it provides evidence for the existence of such source or sources. In our studies, I/O was always > 1. We have noted that the value of the ratio depends on the season, bioaerosol fraction, and animal species. I/O took values from 3 for rooms for colobinae (spring, TC) to 785 for shelters for elephants (spring, RF) (Table 5).

Besides bioaerosols, animal shelters are polluted with dust. There is strong dependence between the two, as airborne microbes are usually associated with dust particles (Zhai et al. 2018). The concentration of solids in facilities intended for animals correlates mainly with the type of animal, its activity, age, density as well as housing system (shallow litter, deep litter, no litter), type of feed, and environmental factors (season, time of the day, humidity in the rooms). The dust in animal facilities comes from different sources, mainly from litter, animal skin and fur, fodder, feces, urine, insect parts, molds, and pollen. We can assume that dust from facilities housing zoo animals may be transferred to different places on shoes and clothing used by animal keepers, what constitutes threat to other people.

Pursuant to the Regulation of the Minister of the Environment, the maximum daily concentration for PM10 fraction in Poland amounts to 0.05 mg/m^3^ (Journal of Laws 2012). It must be pointed out that the threshold value was established for the atmospheric air, while such value was not determined for dust concentration inside facilities. It is worth mentioning that during the heating season (autumn and winter), the threshold for PM10 is regularly exceeded in many towns in Poland (Reizer and Juda-Rezler 2016; European Environment Agency 2017).

In our study, the concentration of PM10 inside animal shelters (Table 6) varied insignificantly. The maximum average daily concentration for PM10 was exceeded only in the winter in all animal facilities and the control area (from 32 to 124%). It must be highlighted that PM10 concentration in animal shelters was lower than the one measured within the control area. Similar relationship was discovered by Reizer and Juda-Rezler (2016). The exposure limits for the fraction of organic dust of animal and plant origin amounting to 4 mg/m^3^ for total dust content and to 2 mg/m^3^ for the respirable fraction were defined in the Regulation of the Polish Minister of Family, Labor and Social Policy (2017). Neither of the values mentioned above was exceeded in our study. The literature on the concentration of dust in animal rooms in zoological gardens is very scarce. In the zoo in Kraków, dustiness reaches similar values (Grzyb and Lenart-Boroń 2019). The comparison of the concentrations recorded in our study with values obtained for livestock rooms revealed that the latter ones are significantly more polluted. On poultry farms, PM10 concentration may reach the values ranging from 0.73 to 15.2 mg/m^3^ (Cambra-López et al. 2010; Viegas et al. 2013). Cambra-López et al. (2010) showed that in swine houses, these values are in the range between 0.05 and 15.3 mg/m^3^. Very high maximum concentrations for the respirable fraction reaching almost 60 mg/m^3^ were observed in cowsheds (Samadi et al. 2013).

In the case of rooms intended for animals, it is not possible to directly assess animal thermal comfort. We can only measure microclimatic parameters that affect this condition, such as temperature, humidity, and air movement (Tombarkiewicz et al. 2008). The temperatures recommended for animals in zoos range between 16 and 30 °C (Kołacz and Dobrzański 2006). In the present study, the temperatures recorded in animal shelters ranged from 16.0 to 23.2 °C (Table 6). The lowest temperature was recorded in the facilities for camels, while the highest for elephants. The temperatures obtained here were within the optimal range for these animals. The negative correlation between the temperature and PM10 concentration can result from the fact that when the outside temperature is high enough, the animals go out on the paddocks. In the case of livestock facilities, indoor temperatures are usually lower, especially during cold periods and are within the range of 10–15 °C. According to Bombik et al. (2009), these temperatures may drop to few degrees. The temperatures recorded by Matković et al. (2006) in the studies undertaken in the autumn in cowsheds ranged between 11.2 and 13.1 °C. In the present experiment, the temperature similar to the one referred above was noted only in the shelters for camels.

As reported by Matković et al. (2006), temperature and relative humidity have direct impact on the concentration and viability of bacteria contained in bioaerosol. Islam et al. (2019) demonstrated that the activity of bacterial bioaerosol in the air is reduced when the relative humidity is too low, what results from suppressing microbe metabolism and physical activity. It has also been shown that Gram-positive bacteria are able to survive longer in the air under high humidity conditions, while Gram-negative bacteria prefer lower humidity (Islam et al. 2019).

Maintaining comfortable temperature in animal shelters is a lot easier than keeping proper relative humidity (RH) of the air. The following factors affect the amount of moisture in the air: evaporation from the animal skin, animal excrement, especially urine, wet floor and walls, type of fodder, and outside air supply. The value of humidity recommended for various types of animals should be in the range between 50.0 and 80.0% (Kołacz and Dobrzański 2006). Too high humidity inside facilities intended for animals creates favorable conditions for the development of undesired microflora and is responsible for faster body cooling. On the other hand, too low humidity affects the condition of animal skin and mucous membranes and leads to excessive dryness and cracking, what in turn increases the risk of developing infection. In our experiment, the average humidity recorded in animal shelters (Table 6) was within the recommended range. Relative humidity above 80% was noted only once in facilities housing colobinae and camels. Relative humidity observed by Islam et al. (2020) in the studies conducted in shelters for calves was similar to the ones recorded in our experiment. The average relative humidity measured by Matković et al. (2006) and Islam et al. (2019) in cowsheds was slightly below 80%.

A negative relationship between the air movement and the concentration of particles from bacterial aerosol fractions with a diameter above 4.7 μm was also found. It seems that the aerosol particles were carried out with the air flow outside the animal shelters.

The time spent by animals in their premises has a significant impact on the level of bacterial bioaerosol. In the cold period of the year, animals stay indoors for longer, and some of them do not leave the premises at all. This relationship is visible in Table 5. The highest values of the I/O ratio for both TC and RF fractions of bacterial aerosol were recorded mainly in the spring and autumn—especially for giraffes and elephants. As mentioned above, a major problem in premises is the relative air humidity, which is the highest during these periods. In the winter, humidity drops, so the I/O ratios are lower. Dust pollution levels also increase during long stay of animals indoors. The dustiness comes mainly from the litter, bacteria from the skin and fur of animals have an equally large share, and other sources are feces and urine, fodder remains. The concentration of bacteria in the air of premises also depends on the size of the animals, their physical activity, and animal density. In our research, we observed a relationship between litter or its absence and the concentrations of bacterial bioaerosol; e.g., kangaroos with litter in their rooms have a high I/O ratio all year round, even in the summer (Table 5), while in colobinae premises without litter, they have a low coefficient I/O all year round. Ropek and Frączek (2016) obtained results similar to the above results.

As referred to above, there are no comprehensive studies regarding the microbiology and microclimate inside animal shelters in zoos. Current findings will provide new information on the concentration of bacteria, particulate level, and zoohygienic conditions. The results obtained in this study will enable implementation of activities aimed at reducing potential threats posed by bacteria to zoo employees and visitors. However, we must be aware that the application of culture-based method has certain limitations. According to some reports, culturable airborne microorganisms constituted as little as 0.5–3.9% of the total identified microflora (Harkawy et al. 2011). This study should be followed by a qualitative analysis of bioaerosol that will allow to provide detailed classification of harmful agents present in such facilities and the assessment of the risk to zookeepers and visitors health.

## Conclusions

Our study showed significant differences between the facilities for various animals in terms of the concentrations of bacterial bioaerosol components. The level of bacterial bioaerosol including the share of respirable fraction is the most significantly determined by the size of the animals and the type of litter the animals have in their shelters.

Concentrations of airborne bacteria were relatively low and in almost all cases did not exceed the limit values proposed by the Polish authorities with respect to the environmental exposure of humans. Bioaerosol levels observed in our study should not be harmful to the health of the zoo workers or its visitors; however, the share of respirable fraction may possibly pose a threat. In most investigated animals, the respirable fraction was leading, with the mean share amounting to approximately 70% of the total fraction of bioaerosol. It means that special attention should be paid to thorough cleaning of the enclosures for animals and animals itself. Maintaining appropriate levels of microclimatic parameters in the shelters is equally important.

The results obtained in this study are a continuation of the research conducted previously at the zoo in Krakow. They extend knowledge about the threats arising from the presence of bioaerosol in animal enclosures. This matter is extremely important because the zoos are often and willingly visited by entire families with young children. Young children are more prone to react to bioaerosol. Moreover, zoo employees who have direct contact with animals are also exposed to bacterial aerosol.

Our study can be an important contribution and provide basic information to decision makers issuing indoor air quality standards for typical bacterial aerosol concentrations in zoo facilities. Zoo managers can use the information obtained in this study, which clearly indicates that ventilation in animal rooms should be modified to improve the conditions for employees and visitors and of course animals.

## Data Availability

The datasets used and/or analyzed during the current study are available from the corresponding author on reasonable request.
